# Apical ballooning syndrome complicated by acute severe mitral regurgitation with left ventricular outflow obstruction – Case report

**DOI:** 10.1186/1749-8090-2-14

**Published:** 2007-02-21

**Authors:** Manju D Chandrasegaram, David S Celermajer, Michael K Wilson

**Affiliations:** 1 Department of Cardiothoracic Surgery, Royal Prince Alfred Hospital, Sydney, Australia; 2 Department of Cardiology, Royal Prince Alfred Hospital, Sydney, Australia

## Abstract

**Background:**

Apical ballooning syndrome (or Takotsubo cardiomyopathy) is a syndrome of transient left ventricular apical ballooning. Although first described in Japanese patients, it is now well reported in the Caucasian population. The syndrome mimicks an acute myocardial infarction but is characterised by the absence of obstructive coronary disease. We describe a serious and poorly understood complication of Takotsubo cardiomyopathy.

**Case Presentation:**

We present the case of a 65 year-old lady referred to us from a rural hospital where she was treated with thrombolytic therapy for a presumed acute anterior myocardial infarction. Four hours after thrombolysis she developed acute pulmonary oedema and a new systolic murmur. It was presumed she had acute mitral regurgitation secondary to a ruptured papillary muscle, ischaemic dysfunction or an acute ventricular septal defect.

Echocardiogram revealed severe mitral regurgitation, left ventricular apical ballooning, and systolic anterior motion of the mitral valve with significant left ventricular outflow tract gradient (60–70 mmHg). Coronary angiography revealed no obstructive coronary lesions.

She had an intra-aortic balloon pump inserted with no improvement in her parlous haemodynamic state. We elected to replace her mitral valve to correct the outflow tract gradient and mitral regurgitation. Intra-operatively the mitral valve was mildly myxomatous but there were no structural abnormalities. She had a mechanical mitral valve replacement with a 29 mm St Jude valve. Post-operatively, her left ventricular outflow obstruction resolved and ventricular function returned to normal over the subsequent 10 days. She recovered well.

**Conclusion:**

This case represents a serious and poorly understood association of Takotsubo cardiomyopathy with acute pulmonary oedema, severe mitral regurgitaton and systolic anterior motion of the mitral valve with significant left ventricular outflow tract obstruction. The sequence of our patient's presentation suggests that the apical ballooning caused geometric alterations in her left ventricle that in turn led to acute and severe mitral regurgitation, systolic anterior motion of the mitral valve and left ventricular outflow tract obstruction. The left ventricular outflow tract obstruction and mitral regurgitation were corrected by mechanical mitral valve replacement. We describe a variant of Takotsubo cardiomyopathy with acute mitral regurgitation, systolic anterior motion of the mitral valve leaflet and left ventricular outflow tract obstruction of a dynamic nature.

## Background

Apical ballooning syndrome (or Takotsubo cardiomyopathy) is a syndrome of transient left ventricular apical ballooning. It was first described in Japanese patients and is now well reported in the Caucasian population. The syndrome mimicks an acute myocardial infarction but is characterised by the absence of obstructive coronary artery disease. We describe a serious and poorly understood complication of Takotsubo cardiomyopathy.

## Case Presentation

We present the case of a 65 year-old lady referred to us from a rural hospital where she was treated with thrombolytic therapy for a presumed acute anterior myocardial infarct. She had presented with central chest pain radiating down her left arm with ECG findings of 2 mm ST elevation in V2 and V3. There was no recent history of psychological stress although she had been on antidepressant drugs for two years. Four hours after thrombolysis she developed acute pulmonary oedema and a new systolic murmur. At this stage it was presumed she had acute mitral regurgitation secondary to a ruptured papillary muscle, ischaemic dysfunction or an acute ventricular septal defect.

**Figure 1:**
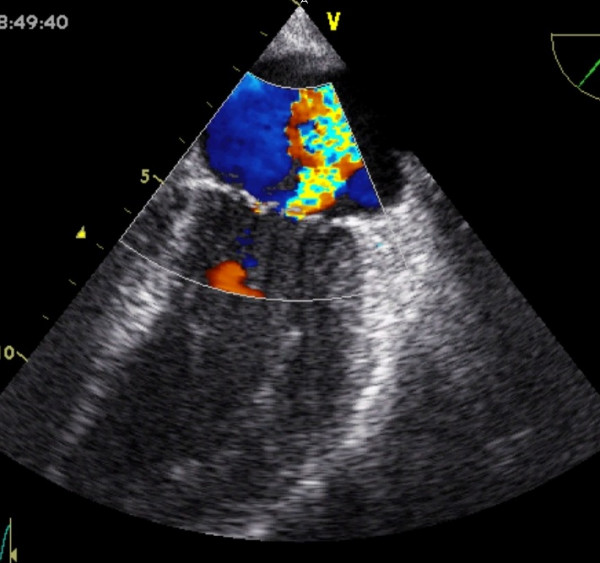
Mitral regurgitation.

**Figure 2:**
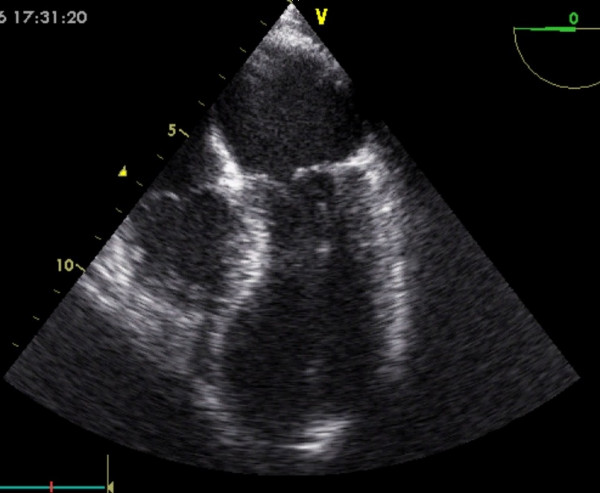
Left Ventricular Apical Ballooning (LVAB).

**Figure 3:**
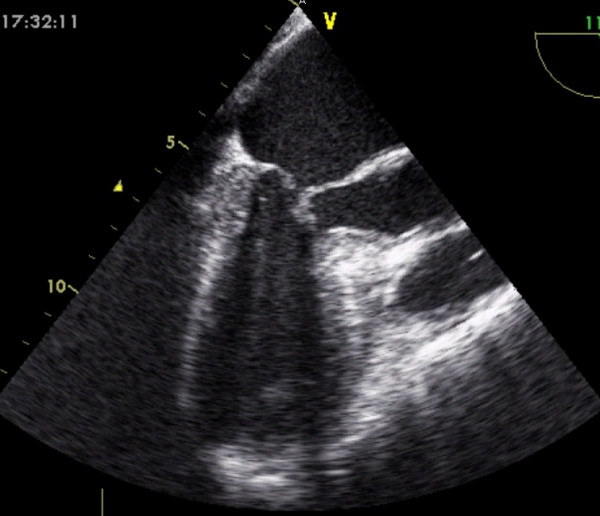
Systolic motion of the anterior leaflet of the mitral valve (SAM).

**Figure 4:**
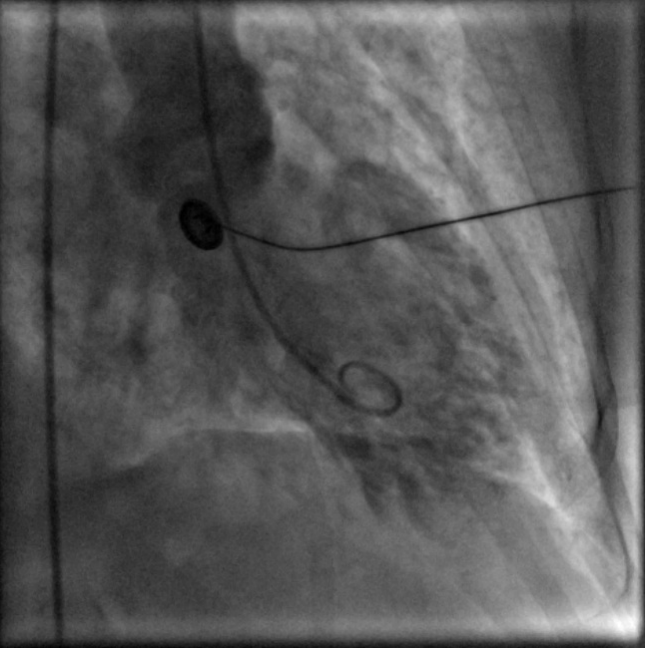
Ventriculogram (Diastole).

**Figure 5:**
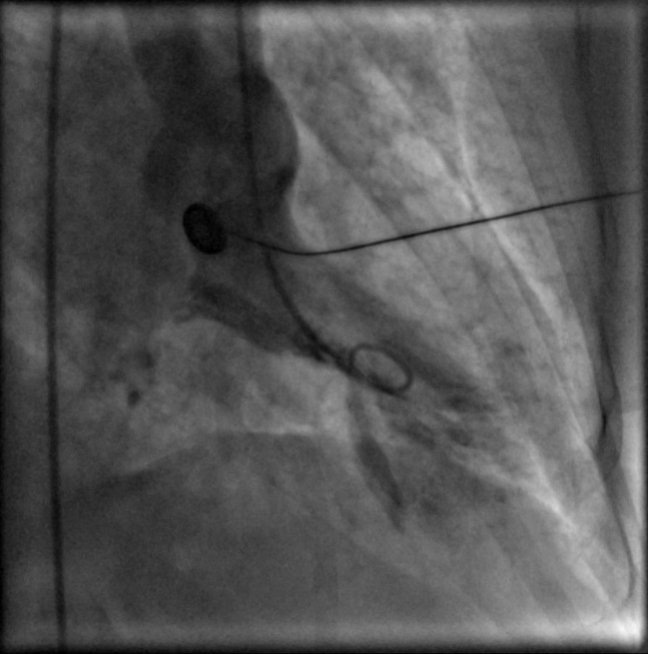
Ventriculogram (Systole).

She was transferred on heparin and glyceryl trinitrate infusion, in acute pulmonary oedema (confirmed by chest X-ray), with a systolic blood pressure of 110 mmHg, and heart rate of 130/minute. Urgent echocardiogram showed severe mitral regurgitation (see Figure 1). Her mitral valve annulus was slightly widened at 3.2 cm and subvalvular structures were intact. She also had left ventricular apical ballooning (LVAB) and systolic anterior motion (SAM) of the mitral valve leaflet with septal contact – left ventricular outflow tract gradient (LVOT) 60–70 mmHg (see Figures 2 and 3). Coronary angiography fourteen hours after her presentation showed no obstructive coronary lesions. Ventriculogram revealed akinesis of the anterolateral wall and apex of her left ventricle, more extensive than any single coronary territory, and grade 4/4 mitral regurgitation (see Figures 4 and 5).

She had an intra-aortic balloon pump (IABP) inserted and was transferred to theatre shortly thereafter. She was in cardiogenic shock, with severe MR and SAM still prominent, despite the IABP. We elected to replace her mitral valve, and at operation, the mitral valve was mildly myxomatous but there were no structural abnormalities, chordae and papillary muscles being intact. She had a mechanical mitral valve replacement (MVR) with a 29 mm St Jude valve. The anterior leaflet was excised and PTFE (gore-tex CV4) was used to reconstruct the subvalvular apparatus. The posterior leaflet was plicated to the annulus. Post-operatively there was no LV outflow obstruction and ventricular function improved to within normal limits by the 10^th ^post-operative day. She recovered well.

## Discussion

Apical ballooning syndrome (or Takotsubo cardiomyopathy) is a syndrome of transient left ventricular apical ballooning which resembles a Japanese octopus trap ("tako-tsubo"). It was initially described in Japanese patients and was thought to be secondary to multivessel coronary spasm [1,2]. The syndrome mimicks an acute myocardial infarction but is characterised by the absence of obstructive coronary artery disease. In a large series of 88 patients with the syndrome, LV function was abnormal at presentation but improved rapidly from 41± 11% (range: 10–62%) to 64 ± 10% (range: 44% to 88%, p < 0.0001) in a period of 21 ± 11 days (range: 14 to 46 days) [3]. There have been increasing reports of this syndrome in Caucasians, with the majority of patients being post-menopausal women (82–100%, mean age 62 to 75 years) [4]. Several reports have found that preceding psychological stresses act to trigger the syndrome [4-6].

Our patient had a complex presentation with echocardiogram revealing extensive anteroapical akinesis, SAM and MR. The sequence of our patient's presentation suggests that the apical ballooning caused geometric alterations in her LV that in turn led to acute and severe MR, SAM and LVOT obstruction. Despite maximal medical therapy including IABP placement, the SAM persisted and probably prevented the usual recovery seen in this syndrome. Intra-operatively, there was no obvious acute alteration in the anatomical structure of her mitral valve, but given the degree of MR and haemodynamic collapse despite IABP, she had a mechanical MVR.

## Conclusion

This case represents a serious and poorly understood association of Takotsubo cardiomyopathy with acute pulmonary oedema, severe MR and SAM with significant LVOT gradient. The sequence of presentation suggests that the apical ballooning caused geometric alterations in the LV that in turn led to acute and severe MR, SAM and LVOT obstruction. We describe a variant of Takotsubo cardiomyopathy with acute MR, SAM and LVOT obstruction of a dynamic nature.

## List of Abbreviations

ECG – Electrocardiogram

IABP – Intra-aortic balloon pump

LV – Left ventricle

LVAB – Left ventricular apical ballooning

LVOT – Left ventricular outflow tract

MR – Mitral regurgitation

MVR – Mitral valve replacement

SAM – Systolic motion of the anterior mitral valve leaflet

## Competing interests

The author(s) declare that they have no competing interests.

## Authors' contributions

MC cared for the patient and drafted the manuscript.

DS cared for the patient, performed the investigations that led to the patient's diagnosis and assisted in the formulation of the manuscript.

MW cared for the patient, performed the operation, prepared the images for publication and assisted in the formulation of the manuscript

All authors read and approved the final manuscript.

## References

[B1] Satoh H, Tateishi H, Uchida T, Dote K, Ishihara M, Kodama K, Haze K, Hon M (1990). Stunned myocardium with specific (tsubo-type) left ventriculographic configuration due to multivessel spasm. Clinical Aspects of Myocardial Injury: From Ischemia to Heart Failure.

[B2] Dote K, Sato H, Tateishi H, Uchida T, Ishihara M, Yoshimura M, Ohkura Y, Watanabe M, Muraoka Y (1991). Myocardial stunning due to simultaneous multivessel coronary spasms: A review of 5 cases [in Japanese]. J Cardiol.

[B3] Tsuchihashi K, Ueshima K, Uchida T, Oh-mura N, Kimura K, Owa M, Yoshiyama M, Miyazaki S, Haze K, Ogawa H, Honda T, Hase M, Kai R, Morii I (2001). Transient Left ventricular apical ballooning without coronary artery stenosis: a novel heart syndrome mimicking acute myocardial infarction. J Am Coll Cardiol.

[B4] Bybee KA, Kara T, Prasad A, Lerman A, Barsness GW, Wright S, Rihal CS (2004). Systematic Review: Transient Left Ventricular Apical Ballooning: A Syndrome That Mimics ST-Segment Elevation Myocardial Infarction. Annals of Internal Medicine.

[B5] Brandspiegel HZ, Marinchak RA, Rials SJ, Kowey PR (1998). A broken heart. Circulation.

[B6] Sharkey SW, Lesser JR, Zenovich AG, Maron MS, Lindberg J, Longe TF, Maron BJ (2005). Acute and reversible cardiomyopathy provoked by stress in women from the United States. Circulation.

